# Air Pollution and Human Health: A Comment from the World Health Organization

**DOI:** 10.5334/aogh.2712

**Published:** 2019-12-16

**Authors:** Maria P. Neira

**Affiliations:** 1 Department of Public Health, Environmental and Social Determinants of Health at the World Health Organization, Geneva, CH

The latest publication from the five National Academies of Sciences of South Africa, Brazil, Germany and the United States and the US National Academy of Medicine on air pollution and health only adds to the growing body of evidence that air pollution is a global public health emergency. This is undoubtedly a different kind of “emergency” from the one that we are accustomed to dealing with in public health. In fact, it is hardly ever really unexpected and it is not an unforeseen occurrence, an accident or a turn of events; it is the result of many different decisions that have produced an emergency.

According to WHO, air pollution is responsible for 7 million premature deaths yearly from exposure to ambient and household air pollution that lead to diseases such as stroke, heart disease, lung cancer, chronic obstructive pulmonary diseases and respiratory infections, including pneumonia. The costs of air pollution to society and the economies of low- and middle-income countries are enormous, quantifiable in terms of percentage of gross domestic products (GDPs).

The National Academies have succinctly and accurately summarized the current understanding and the urgent need for a call to action. We all need to tackle the persistent global scourge of air pollution, its transboundary and local nature, and its victims both in urban and rural areas. I fully commend and support the adoption of a global compact on air pollution to make air pollution control and reduction a priority for all. By calling on governments, leaders, businesses and citizens to take urgent action on reducing air pollution throughout the world to the benefit of human health and well-being, and to benefit the environment and as a condition towards the cross-cutting Sustainable Development Goals. The development of new forms of information and knowledge are a challenge and an opportunity to reinforce alliances, discussions and evidence able to counteract trends of pseudoscience, misinformation and fabrication of dangerous narratives. The science keeps moving on, yet the list of maladies linked to air pollution continues to grow. We need to build instruments that can structure new public health “weapons” that raise awareness, communicate the scientific debate, indicate solutions and mobilize resources and strategies. The adoption of the global compact would ensure sustained engagement at the highest level and would provide a further peg for policy makers and other key partners, including the private sector, to integrate emission control and reduction into national and local planning, development processes, and business and finance strategies. Political leadership, partnerships and multinational structures are essential.

WHO stands ready to bolster efforts to support countries to reduce air pollution and protect health and looks forward to a strong collaboration with the Academies to provide decision makers with specific information on which interventions are most effective in improving air quality and health. We have 7 million reasons to do it.

WHO applauds this initiative by the National Academies of Science and Medicine.

